# N*ε*-Carboxymethyl-Lysine Negatively Regulates Foam Cell Migration via the Vav1/Rac1 Pathway

**DOI:** 10.1155/2020/1906204

**Published:** 2020-02-28

**Authors:** Zhengyang Bao, Lili Zhang, Lihua Li, Jinchuan Yan, Qiwen Pang, Zhen Sun, Yue Geng, Lele Jing, Chen Shao, Zhongqun Wang

**Affiliations:** ^1^Department of Cardiology, Affiliated Hospital of Jiangsu University, Zhenjiang 212001, China; ^2^Department of Internal Medicine, Affiliated Wuxi Maternity and Child Health Care Hospital of Nanjing Medical University, Wuxi 214000, China; ^3^Department of Pathology, Affiliated Hospital of Jiangsu University, Zhenjiang 212001, China

## Abstract

**Background:**

Macrophage-derived foam cells play a central role in atherosclerosis, and their ultimate fate includes apoptosis, promotion of vascular inflammation, or migration to other tissues. N*ε*-Carboxymethyl-lysine (CML), the key active component of advanced glycation end products, induced foam cell formation and apoptosis. Previous studies have shown that the Vav1/Rac1 pathway affects the macrophage cytoskeleton and cell migration, but its role in the pathogenesis of diabetic atherosclerosis is unknown.

**Methods and Results:**

In this study, we used anterior tibiofibular vascular samples from diabetic foot amputation patients and accident amputation patients, and histological and cytological tests were performed using a diabetic ApoE^−/−^ mouse model and primary peritoneal macrophages, respectively. The results showed that the atherosclerotic plaques of diabetic foot amputation patients and diabetic ApoE^−/−^ mice were larger than those of the control group. Inhibition of the Vav1/Rac1 pathway reduced vascular plaques and promoted the migration of macrophages to lymph nodes. Transwell and wound healing assays showed that the migratory ability of macrophage-derived foam cells was inhibited by CML. Cytoskeletal staining showed that advanced glycation end products inhibited the formation of lamellipodia in foam cells, and inhibition of the Vav1/Rac1 pathway restored the formation of lamellipodia.

**Conclusion:**

CML inhibits the migration of foam cells from blood vessels via the Vav1/Rac1 pathway, and this process affects the formation of lamellipodia.

## 1. Introduction

The recruitment of macrophages to the intima and phagocytosis of lipids to form foam cells is an essential step in the progression of atherosclerosis [1, 2]. Regarding the fate of foam cells in plaques, the traditional belief is that apoptosis of macrophages further increases plaque inflammation and promotes the progression of atherosclerosis [3]. However, with a deeper understanding of the pathogenesis of atherosclerosis, researchers have gradually realized that the migration of foam cells from arterial wall lesions and the migration of monocytes and macrophages are dynamic processes. The obstruction of foam cell migration is another important factor in the accumulation of foam cells in plaques [4].

N*ε*-Carboxymethyl-lysine (CML) is a major active ingredient in advanced glycation end products. Studies have shown that N*ε*-carboxymethyl-lysine can promote foam cell formation, inhibit foam cell migration, and accelerate the progression of atherosclerosis, but the specific mechanism is not yet clear [5, 6]. Vav1 protein, as a member of the guanine exchange factors (GEFs), affects the activity of the Rho GTPase family through phosphorylation of its specific site [7]. Rahaman et al. and Chen et al. found that the degree of Vav1 phosphorylation was increased in atherosclerotic plaques, while knocking out Vav1 reduced plaque area [8, 9]; in vitro experiments showed that the loss of the Vav1 gene recovered the migratory ability of oxLDL-induced foam cells [10], but whether the migration of foam cells is affected by Vav1 gene expression and phosphorylation in diabetes is not clear.

Members of the Rho GTPase family play an important role in intracellular signaling pathways. Previous studies have shown that the Rho GTPase family can participate in intercellular function changes, including cell adhesion, contraction, proliferation, and migration, by regulating the polymerization state of the cytoskeleton [11]. During cell signal transduction, Rho protein exists in the GTP-binding form (active state) and GDP-binding state (inactive state) and initiates or terminates the cell signal cascade activation reaction through the transition between the two binding states [12]. Studies have shown that Rho protein regulates cell migration by regulating the phosphorylation level of myosin light chain (MLC), but it is not clear whether Rho protein affects foam cell migration during diabetic atherosclerosis [13]. Ras-related C3 botulinum toxin substrate 1 (Rac1), as a member of Rho GTPase super family, plays a central role in cytoskeleton polymerization and migration. This study used in vitro and in vivo experiments to demonstrate that Vav1/Rac1 signaling promotes the progression of atherosclerosis by affecting the migratory ability of foam cells in atherosclerotic plaques.

## 2. Materials and Methods

### 2.1. Human Studies

Anterior tibial arteries from human diabetic amputees (*n* = 4) and from normal amputees (*n* = 4) were obtained from the Affiliated Hospital of Jiangsu University (Zhenjiang, China) from February 2015 to June 2017. This study was approved by the Chinese Clinical Trial Registry and conducted in agreement with the institutional guidelines. Written informed consent was obtained from all patients. Ethical review number is ChiECRCT20190206.

### 2.2. Animal Studies

The experimental protocols were approved by the Institutional Animal Care and Use Committee of Jiangsu University (Jiangsu, China) and performed in accordance with the Guidelines for Animal Experimentation of the National Institutes of Health. Male ApoE^-\-^ mice with a C57BL/6J background were obtained from the Jackson Laboratory (USA). The diabetic ApoE^-\-^ model was established as described previously. In brief, at 6 weeks of age, the mice were rendered diabetic through intraperitoneal injection for five consecutive days of 40 mg/kg STZ dissolved in 100 mM citrate buffer (pH 4.5). Mice with blood glucose levels of ≥300 mg/dL after 2 weeks of STZ administration were considered diabetic.

### 2.3. Cell Culture

Peritoneal macrophages (PM*φ*) were harvested from C57BL/6 mice 4 days after thioglycollate injection by peritoneal lavage as described. Isolated PM*φ* were plated on glass coverslips. After 1 hour of incubation at 37°C, nonadherent cells were removed by gentle washing, and the remaining cells were cultured overnight in DMEM containing 10% FBS before use in the phagocytosis assay.

The Raw 264.7 macrophage cell line was purchased from the American Type Culture Collection and cultured in DMEM medium supplemented with 10% FBS, 2 mM L-glutamine, 100 U/mL penicillin, and 100 *μ*g/mL streptomycin at 37°C in a humidified atmosphere with 5% CO_2_. A foam cell model was established by using Raw 264.7 macrophages loaded with 40 *μ*g/mL oxLDL.

### 2.4. Oil Red O Staining

Atherosclerotic lesions in the aorta or para-aortic lymph nodes of ApoE^-\-^ mice were subsequently stained with freshly filtered Oil Red O working solution for 30 min at room temperature, and then, the staining was evaluated under an inverted microscope (Olympus, IX51).

### 2.5. In Vivo Studies of Macrophage Migration from the Peritoneum

The mice (*n* = 4/group) were injected intraperitoneally with 3% thioglycollate to trigger sterile peritonitis. After 4 days, peritoneal macrophages were labeled by injection of 1 *μ*m Fluoresbrite green fluorescent plain microspheres (Polysciences). On the next day, the mice were injected with an inflammatory stimulus (400 ng LPS) to induce efflux of macrophages from the peritoneum to the draining lymph nodes. Following LPS injection (3 hours), the percentage of macrophages in the lavage fluid was quantified by flow cytometry using the PE/Cy7-conjugated anti-mouse F4/80 antibodies (BioLegend).

### 2.6. Transwell Migration Assay

PM cells suspended in DMEM containing 1% FBS were placed in a 24-well Transwell migration chamber and treated with oxLDL and CML. DMEM containing 10% FBS was added into the lower chambers. Afterwards, the medium from the upper chamber was carefully aspirated. The membrane was fixed with 4% PFA for 30 min and stained with 0.1% crystal violet for 20 min. Migration of Raw 264.7 cells was evaluated by viewing under high-power magnification and counting the migrated cells in six randomly selected fields/well viewed.

### 2.7. Wound Healing Assay

PM cells were seeded in a 6-well culture plate and grown to 80–85% confluence; subsequently, a scratch was made through the cell layer by using a sterile micropipette tip. The wounded areas were photographed under a light microscope. Cell migration was assessed by measuring the size of the scratch area.

### 2.8. Immunofluorescence Staining

Isolated aortas and para-aortic lymph nodes were placed in 10% neutral buffered formalin overnight, embedded in paraffin, and sliced into 4 *μ*m thick sections. Atherosclerotic lesions in the aorta or the para-aortic lymph nodes were shown by Oil Red O staining. The distribution and expression levels of CD68 were estimated by immunofluorescence staining. The mean fluorescence density of positive cells was determined as the optical density/area by ImageJ. Semiquantitative analysis of immunofluorescence staining of CD68 was determined as the mean fluorescence density of CD68/DAPI.

### 2.9. Western Blot Assay

Raw 264.7 cells, isolated aortas, or para-aortic lymph nodes were washed with cold PBS and incubated in lysis buffer (RIPA) containing 1 mmol/L PMSF protease inhibitor (Sigma, St. Louis, MO, USA) and phosphatase inhibitors (Invitrogen, Carlsbad, CA, USA) on ice and then centrifuged at 12,000 g and 4°C for 15 min. Protein concentrations were determined using a micro BCA protein assay kit (Thermo Fisher Scientific, Rockford, USA). Protein samples were loaded onto a polyacrylamide gel and transferred onto PVDF membranes by using a semidry method. The membranes were blocked in 5% milk in TBST, followed by overnight incubation with primary antibodies. The membranes were subsequently washed with TBST and then incubated with HRP-conjugated secondary antibodies. Immunoreactive bands were visualized by chemiluminescence (ECL Amersham Pharmacia). Protein expression levels were quantified by densitometric analysis by using LANE 1D software.

### 2.10. Statistical Analysis

Data are presented as the mean ± S.D., and SPSS 17.0 software was used to analyze the data. Comparisons between two variables were analyzed using the unpaired Student's *t*-test. Comparisons among multiple treatment groups were assessed by one-way ANOVA, followed by a post hoc LSD test. *P* < 0.05 was considered statistically significant.

## 3. Results

### 3.1. Vav1 Expression Is Elevated in the Vasculature of Diabetic Foot Amputation Patients, while Vav2 and Vav3 Expressions Are Not Elevated

**Figure 1:**
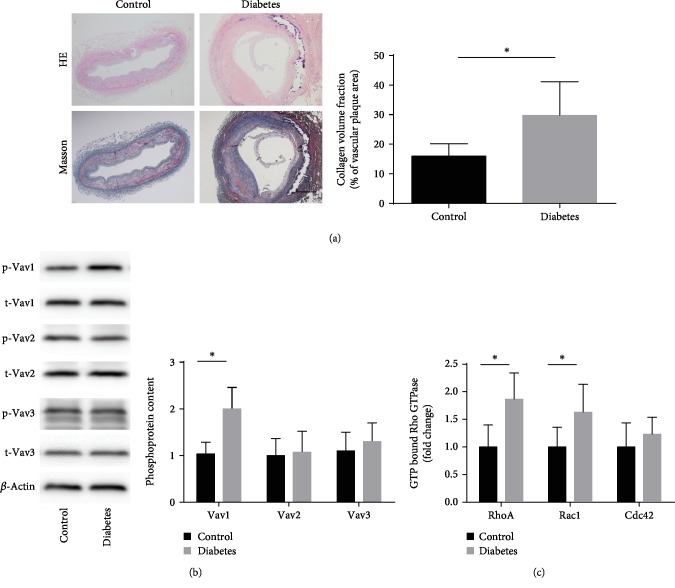
Phosphoprotein of Vav1 and activity of RhoA and Rac1 increase in diabetes amputees. (a) Representative images of anterior tibial artery sections stained with Masson/hematoxylin from accident amputees and diabetic amputees; scale bars, 50 *μ*m. (b) The phosphoprotein content of Vav was detected by Western blot. (c) The activity of GTP-bound GTPase was detected by G-Lisa; values are presented as the mean ± SD from three independent experiments. ^∗^*P* < 0.05.

Anterior tibial arteries from diabetic and control amputees (accident amputation) were obtained from the Affiliated Hospital of Jiangsu University. Masson's staining revealed that the fiber cap of atherosclerosis lesion in diabetic amputees was thinner than that in control amputees (Figure 1(a)). We performed Western blot analysis to evaluate the degree of phosphorylation of Vav family members in the lower extremity anterior tibial arteries of patients with diabetic foot amputation and of patients with car accident amputation. The results showed that the degree of Vav1 phosphorylation was significantly increased in patients with diabetic amputation, while there was no significant difference in the degree of phosphorylation of Vav2 and Vav3 compared with that of the control group (Figure 1(b)).

### 3.2. The Activity of Rac1 and RhoA Increased in the Vasculature of Diabetic Foot Amputation Patients

The Vav family acts as important guanine exchange factors (GEFs) that regulate the activity of small GTPases. By regulating the binding and dissociation of small GTPases and GTP in atherosclerosis, we detected intravascular small GTPase activity in patients with diabetic foot amputation and accident amputation. The results showed that enzymatic activity of the small GTPase family members Rac1 and RhoA (with GTP-binding state) increased, but the expression of another small GTPase, Cdc42, did not change significantly (Figure 1(c)).

### 3.3. Interfering with the Expression of the Vav1 Reduces the Accumulation of Foam Cells in the Plate and Promotes Foam Cell Migration to Paravascular Lymph Nodes

**Figure 2:**
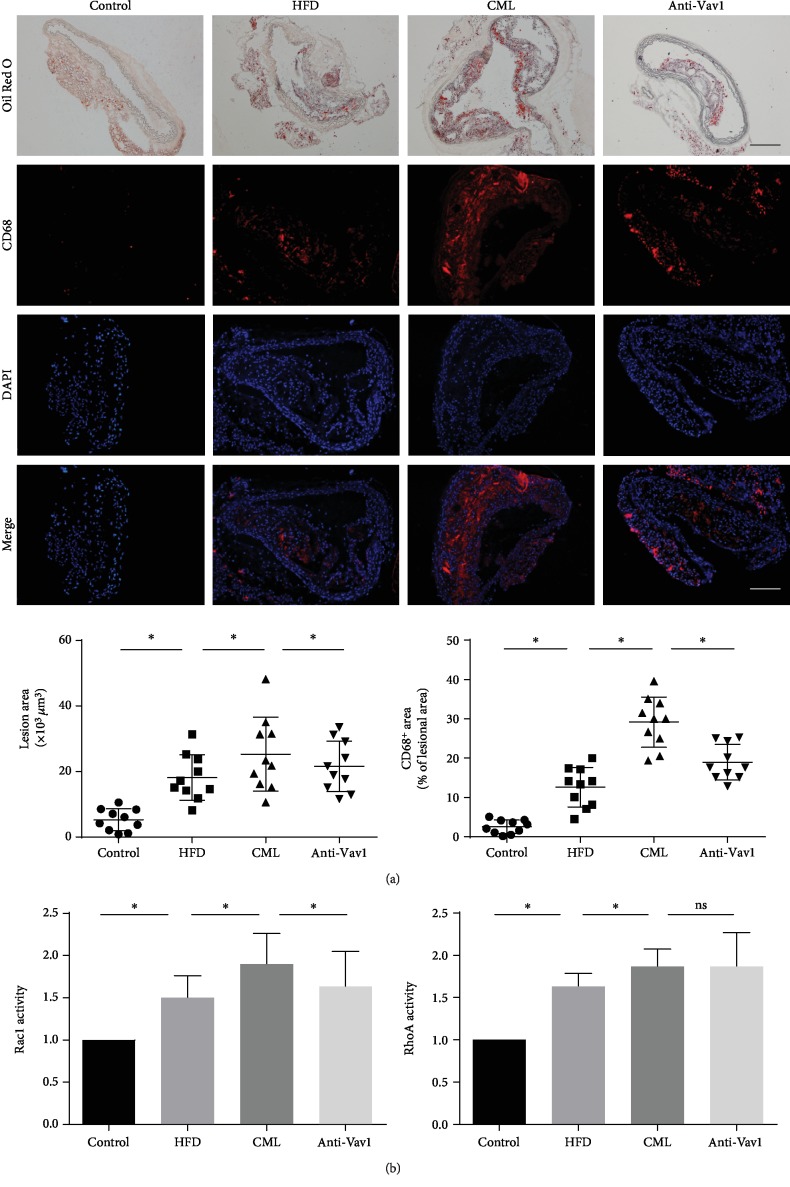
Inhibition of Vav1 expression antagonizes the effect of CML on lipid accumulation in diabetic ApoE^−/−^ mice. (a) ApoE^−/−^ mice were fed with an HF diet and treated with CML and/or anti-Vav1 for 12 weeks. Representative images of aortic root stained with Oil Red O and immunofluorescence. Scale bars, 20 *μ*m. (b) The activity of GTP-bound GTPase was detected by G-Lisa; values are presented as the mean ± SD from three independent experiments. ^∗^*P* < 0.05.

**Figure 3:**
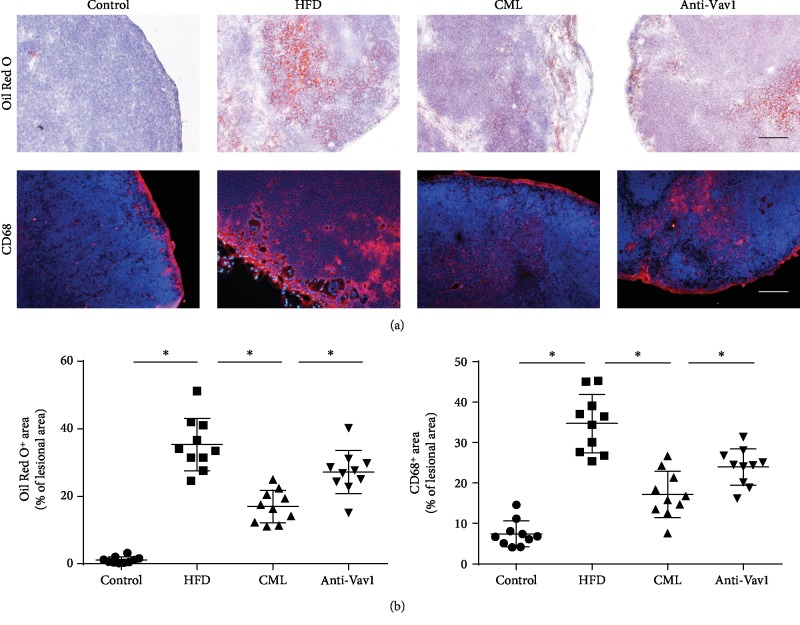
CML inhibits foam cells migrating to the para-aortic lymph node, inhibiting Vav1 reverse macrophage migration ability. (a) Oil Red O staining and immunofluorescence staining for CD68 in the para-aortic lymph node. Scale bars, 20 *μ*m. (b) The extent of Oil Red O-positive area and CD68-positive area in the para-aortic lymph node; values are presented as the mean ± SD from three independent experiments. ^∗^*P* < 0.05.

We established a diabetic ApoE^−/−^ mouse model based on previous methods in our group and administered Vav1 antibody to ApoE^−/−^ mice. Oil Red O staining and CD68 immunogold staining showed increased lipid accumulation in the plaques of high-fat diet-fed ApoE^−/−^ mice, increased foam cells in plaques, vascular plaque area in diabetic ApoE^−/−^ mice, and lipid accumulation in foam cells. The ApoE^−/−^ mice fed with a high-fat diet had further elevation of these factors, and the antibody interfered with the expression of Vav1 (Figure 2(a)). The paravascular lymph nodes of each group were harvested and stained with CD68 and Oil Red O. The high-fat diet-fed ApoE^−/−^ mice showed increased CD68-positive macrophages in the paravascular lymph nodes, and Oil Red O staining showed increased lipid accumulation in the lymph nodes. In the para-aortic lymph nodes of diabetic ApoE^−/−^ mice, the number of CD68-positive macrophages and lipid accumulation increased than that in the high-fat diet-fed ApoE^−/−^ mice, while macrophage and lipid accumulation were significantly higher in the lymph nodes of diabetic mice treated with the anti-Vav1 antibody than those of the mice that were not administered with the Vav1 antibody (Figures 3(a) and 3(b)). Combined with the above results, we believe that lipid accumulation in plaques of diabetic ApoE^−/−^ mice is associated with decreased foam cell migration, and inhibition of Vav1 expression inhibits plaque progression and lipid accumulation. Lymph node staining results suggest that it may be related to inhibition of foam cell migration.

### 3.4. Vav1 Affects the Effect of Advanced Glycation End Products on Foam Cell Migration but Does Not Affect Intracellular Lipid Accumulation

**Figure 4:**
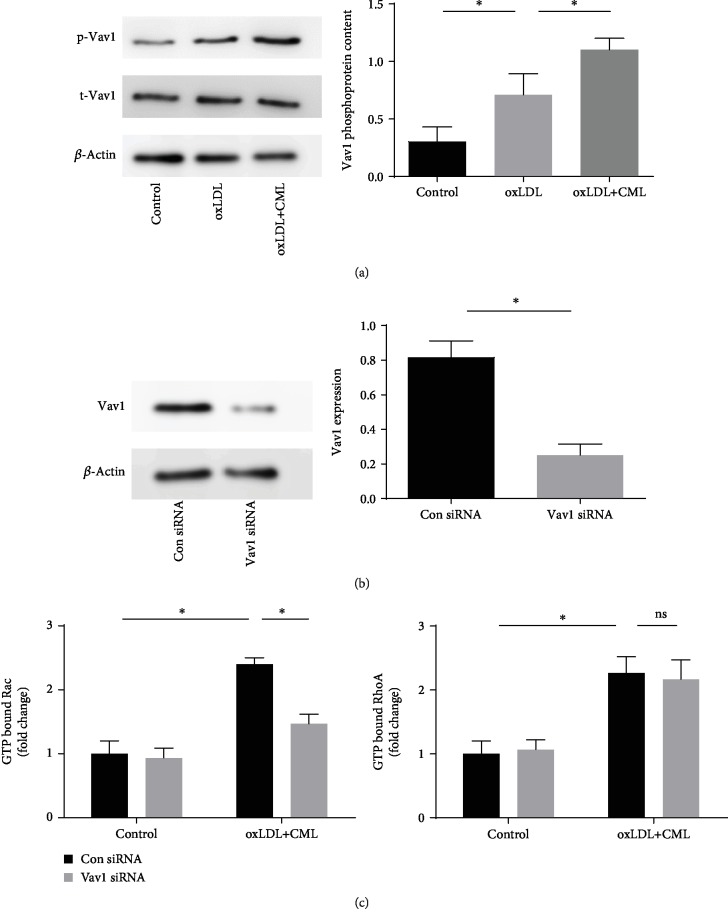
CML increase the phosphorylation of Vav1 in macrophage-derived foam cells, inhibiting Vav1 expression which reduces the activity of Rac1 but has no change on RhoA activity. (a) Phosphorylation of Vav1 was investigated by Western blot. (b) Vav1 siRNA were used to interrupt the expression of Vav1; the effect of Vav1 siRNA was investigated by Western blot. (c) The activity of GTP-bound GTPase was detected by G-Lisa; values are presented as the mean ± SD from three independent experiments. ^∗^*P* < 0.05.

**Figure 5:**
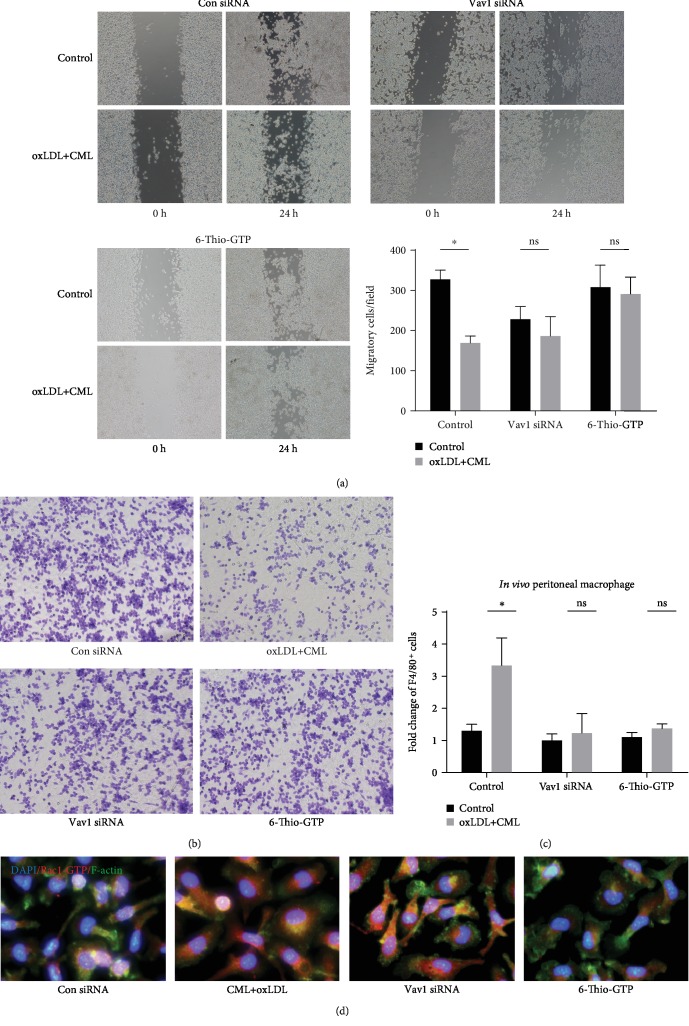
CML inhibit lamellipodia formation and foam cell migration in vivo and in vitro via Vav1/Rac1 pathway. Wound healing assay (a) and Transwell migration assay (b) were used to evaluate the cell migration capacity in vitro (200x magnification). (c) In vivo migration assay: wild type, Vav1 siRNA, and 6-thio-GTP pretreated peritoneal macrophage were treated with oxLDL+CML for 24 h before injected into mice peritoneally, three hours after LPS injection peritoneal cells were collected and the percentage of macrophages in the lavage was quantified by flow cytometry; ^∗^*P* < 0.05. (d) Peritoneal macrophages were exposed to CML+oxLDL in the presence or absence of Vav1 siRNA, and 6-thio-GTP, CD68, Rac1-GTP, and DAPI were stained. Values are presented as the mean ± SD from three independent experiments. Scale bars, 10 *μ*m.

Advanced glycation end products are the end products of diabetic metabolic disorders, and they promote the progression of atherosclerosis by binding to intracellular and extracellular receptors. Western blot analysis revealed that the degree of phosphorylation of Vav1 was significantly increased (Figure 4(a)), and G-Lisa analysis showed that CML increase the activity of RhoA and Rac1 and Vav1 siRNA interference decreased the activity of Rac1 but have no change on RhoA (Figure 4(c)). Transwell migration assays, wound healing assays, and peritoneal macrophage migration assays showed that advanced glycation end products reduced foam cell migration in vivo and in vitro (Figures 5(a)–5(c)). Using Vav1 siRNA to interfere with Vav1 expression, we found that there was no significant change in intracellular lipid accumulation, but foam cell migratory ability was restored in vivo after inhibition of Vav1 expression.

### 3.5. The Vav1/Rac1 Pathway Affects Foam Cell Migration by Regulating Cytoskeletal Separation and Polymerization

We detected the activity of RhoA and Rac1 in foam cells after CML stimulation by G-Lisa assay. The results showed that CML promoted an increase in RhoA and Rac1 activity in foam cells. Inhibition of Vav1 expression by siRNA reduced Rac1 activity, but the change in RhoA activity was not statistically significant. We used 6-thio-GTP, an inhibitor of the Vav1/Rac1 pathway, to pretreat the macrophages, and the results showed that the effect of CML on the migration of foam cells was blocked. Then, we focused on the effect of Vav1/Rac1 on the cell cytoskeleton. Phalloidin staining showed that CML stimulation promoted cytoskeletal spreading and reduced the formation of lamellipodia. After the addition of Vav1 siRNA or 6-thio-GTP, the lamellipodia production was restored (Figure 5(d)), suggesting that the Vav1/Rac1 pathway affects the foam cell cytoskeleton, thereby inhibiting foam cell migration.

## 4. Discussion

Macrovascular complications are the most important lethal and disabling complications in diabetes and are mainly caused by increased oxidative stress, insulin resistance, increased advanced glycation end products, and disorders of glycolipid metabolism [14, 15]. AGEs are involved in microvascular and macrovascular complications through the formation of crosslinks between molecules in the basement membrane of the extracellular matrix and by engaging the receptors for advanced glycation [16, 17]. This study showed that advanced glycation end products inhibited foam cell migration out of the blood vessels by activating the Vav1/Rac1 pathway and reducing the production of lamellipodia.

Excessive accumulation of free cholesterol in macrophages leads to activation of downstream cascades, including the NLRP3 inflammasome, Toll-like receptor signaling, and the endoplasmic reticulum stress response [18]. These inflammatory signals exacerbate not only the oxidative stress in the plaque but also the migration of other inflammatory cells (including monocytes) to the intima [19]. However, reversing atherosclerosis requires the migration of macrophages to plaques and reduction of inflammation. However, during the progression of atherosclerosis, the expression of retention factors prevents macrophages from escaping from the arterial wall [20]. Existing studies have shown that sAGEs can activate monocytes and AGEs deposited on bone marrow macrophages inhibit monocyte migration, inducing a process called “apoptaxis” [21]. In this study, we found that advanced glycation end products inhibit foam cell migration in vascular plaques and promote the progression of atherosclerosis, which illustrates the role of advanced glycation end products in diabetic atherosclerosis.

Cell migration begins with the establishment of a protruding force for membrane extension and traction for contraction [22]. The Rho GTPase superfamily, including Rho, Rac1, and Cdc42, are known to act as molecular switches with their GTP- or GDP-binding forms and are involved in cell migration processes [23, 24]. Recent studies have shown that the guanylate exchange factor Vav family regulates macrophage morphology by regulating Rac1 and RhoA activities [10]. In addition, in atherosclerosis, signals transmitted by oxLDL and CD36 in platelets or macrophages regulate downstream signaling molecules such as MLCK, which affects the cytoskeleton [25]. Our study showed that the phosphorylation of Vav1 is significantly elevated in the process of diabetic atherosclerosis. In vitro experiments showed that hyperphosphorylation of Vav1 inhibits foam cell migration, mainly by regulating the activation of Rac1 but not RhoA. Although recent studies have shown that Vav/Cdc42 regulates actin polymerization to promote low-density lipoprotein uptake and metabolism [26], there is no evidence that Vav/Cdc42 regulates macrophage migration, which may require further research to explore.

The cell migration process involves the formation of lamellipodia, the dissociation of cells from the extracellular matrix, and contraction of the cytoskeletal tail. During this process, Rac promotes the formation of PDGF-stimulated lamellipodia, while RhoA stimulates contractile myogenesis downstream of LPA signaling. Protein fibers (i.e., stress fibers) are formed. CDC42 was later shown to promote and activate Rac, and there is considerable evidence that the activity of Rac maintains the directional front end of protruding lamellipodia [27]. In contrast, Rho is more active at the flank and posterior regions of the cell, antagonizing the function of Rac1 [28]. Staining of F-actin by phalloidin showed that advanced glycation end products inhibited the production of lamellipodia, and lamellipodia production was restored when Vav1/Rac1 was blocked by Vav1 siRNA and 6-thio-GTP. In subsequent experiments, we will use a real-time visualization tool to further investigate the role of the Vav1/Rac1 pathway in the cytoskeleton.

Our data provide additional research support for the final fate and specific matrix of foam cells in diabetes atherosclerosis and validate the role of peritoneal macrophage efflux experiments in foam cell migration studies in diabetic mice, while the role of Vav1/Rac1 in advanced glycation end products inhibiting foam cells needs to be further elucidated. The Vav1/Rac1 pathway inhibits foam cell migration by inhibiting the formation of lamellipodia in macrophages, and blocking each loop of this pathway inhibits the effect of advanced glycation end products. These findings provide further understanding of new ways to promote the regression of atherosclerotic plaques.

## Data Availability

The data used to support the findings of this study are available from the corresponding author upon request.
